# The Effect of Noise-Masking Earbuds (SleepBuds) on Reported Sleep Quality and Tension in Health Care Shift Workers: Prospective Single-Subject Design Study

**DOI:** 10.2196/28353

**Published:** 2022-03-22

**Authors:** Nicole M Duggan, M Adrian Hasdianda, Olesya Baker, Guruprasad Jambaulikar, Andrew J Goldsmith, Anna Condella, Desiree Azizoddin, Adaira I Landry, Edward W Boyer, Andrew J Eyre

**Affiliations:** 1 Department of Emergency Medicine Brigham and Women's Hospital Boston, MA United States; 2 Center for Clinical Investigation Brigham and Women's Hospital Boston, MA United States; 3 Health Promotion Research Center Department of Family and Preventive Medicine University of Oklahoma Health Sciences Center Oklahoma City, OK United States

**Keywords:** shift work, sleep, sleep aid, alertness, earbud, SleepBuds, healthcare worker, physician, health care

## Abstract

**Background:**

Shift work is associated with sleep disorders, which impair alertness and increase risk of chronic physical and mental health disease. In health care workers, shift work and its associated sleep loss decrease provider wellness and can compromise patient care. Pharmacological sleep aids or substances such as alcohol are often used to improve sleep with variable effects on health and well-being.

**Objective:**

We tested whether use of noise-masking earbuds can improve reported sleep quality, sleepiness, and stress level in health care shift workers, and increase alertness and reaction time post night shift.

**Methods:**

Emergency medicine resident physicians were recruited for a prospective, single-subject design study. Entrance surveys on current sleep habits were completed. For 14 days, participants completed daily surveys reporting sleep aid use and self-rated perceived sleepiness, tension level, and last nights’ sleep quality using an 8-point Likert scale. After overnight shifts, 3-minute psychomotor vigilance tests (PVT) measuring reaction time were completed. At the end of 14 days, participants were provided noise-masking earbuds, which they used in addition to their baseline sleep regimens as they were needed for sleep for the remainder of the study period. Daily sleep surveys, post–overnight shift PVT, and earbud use data were collected for an additional 14 days. A linear mixed effects regression model was used to assess changes in the pre- and postintervention outcomes with participants serving as their own controls.

**Results:**

In total, 36 residents were recruited, of whom 26 participants who completed daily sleep surveys and used earbuds at least once during the study period were included in the final analysis. The median number of days of earbud use was 5 (IQR 2-9) days of the available 14 days. On days when residents reported earbud use, previous nights’ sleep quality increased by 0.5 points (*P*<.001, 95% CI 0.23-0.80), daily sleepiness decreased by 0.6 points (*P*<.001, 95% CI –0.90 to –0.34), and total daily tension decreased by 0.6 points (*P*<.001, 95% CI –0.81 to –0.32). These effects were more pronounced in participants who reported worse-than-average preintervention sleep scores.

**Conclusions:**

Nonpharmacological noise-masking interventions such as earbuds may improve daily sleepiness, tension, and perceived sleep quality in health care shift workers. Larger-scale studies are needed to determine this interventions’ effect on other populations of shift workers’ post–night shift alertness, users’ long-term physical and mental health, and patient outcomes.

## Introduction

Over the last several decades, sleep deficiency has reached epidemic proportions. An estimated one-third of adults in the United States do not get the recommended seven hours of sleep nightly, and at least 50 million Americans have chronic sleep disorders [1,2]. Shift work, including working overnight hours, having variable or rotating schedules, or working extended hours on call uniquely contributes to worse sleep and poor work-related outcomes [3-6]. Health care practitioners and support staff represent occupation groups with some of the highest prevalence of shift work and sleep problems including sleep disturbances (eg, multiple awakenings), and sleep deficiency from either extended wake episodes (ie, acute sleep deprivation) or multiple days or nights of insufficient sleep (ie, sleep restriction or chronic sleep deprivation) [7].

In health care workers, sleep loss impairs alertness, which can lead to poor work performance and medical errors, potentially compromising patient care [4,8-11]. Despite the recognized impact of sleep loss on clinician health and patient safety, workplace interventions to combat sleep loss and improve restful sleep are lacking compared to other wellness initiatives [12]. Accordingly, we assessed the impact of noise-masking earbuds on self-reported sleepiness, sleep quality, stress level, and post–overnight shift alertness in emergency medicine (EM) resident physicians.

## Methods

### Study Design and Setting

This prospective, single-subject design study was performed at a single urban academic medical center. This facility hosts residency training programs for multiple specialties including a 4-year EM training program.

### Ethical Considerations

This work was approved by the institutional review board (IRB), and all participants gave informed consent. This study was approved by the Brigham and Women’s Hospital Institutional Review Board (2019p002509).

### Participant Recruitment

EM resident physicians working full-time at the emergency department (ED) during the study period were recruited. All EM residents who were not hearing impaired and who owned a smart phone capable of receiving SMS text messages and opening electronic surveys were eligible for inclusion. Participants were recruited via email to the residency listserv and through a text message in a resident-specific group messaging app delivered to their personal devices.

Within this residency training program, each resident works between 18-21 shifts in a rotating schedule comprising day shifts (roughly 7 AM to 5 PM), twilight shifts (roughly 3 PM to 2 AM), or overnight shifts (roughly 11 PM to 8 AM) over each 28-day period. Each participant works 5-7 overnight shifts over a 28-day period.

### Interventions

Following consent procedures, participants completed an electronic entrance survey that included questions about their baseline sleep habits, sleep aid use, self-reported sleep quality, daily sleepiness, and daily tension over the prior 28 days (Multimedia Appendix 1). Over the next 14 days (the control period), participants were instructed to continue with their baseline sleep habits. On study day 15, each participant was provided with a pair of Bose SleepBuds (Bose Corp). SleepBuds (hereby referred to as “earbuds”) are earbuds worn in both ears all night and function to mask ambient noises by playing various sound tracks selectable from the Bose app, which each target decibels that are specific to common ambient sounds. Participants were given instruction on device functionality including the app for activating the device noise-masking technology, which only occurs when the device is turned on and used in conjunction with the app. Participants were instructed to use the earbuds with the noise-masking technology for their sleep episodes as needed over the next 14 days in addition to their preferred baseline sleep aids. This 14-day period was considered the intervention period. Earbud use was at the discretion of each participant, and study personnel who were not affiliated with the Bose Corporation were available throughout the study period to answer questions about functionality or troubleshoot device function. At the end of the study period, participants completed an electronic exit survey on sleep aid use and earbud functionality over the prior 28-day period (Multimedia Appendix 1).

### Measurements

#### Daily Surveys

Each day of the 28-day study period, participants received an automated SMS text message at 12 noon containing a daily survey asking them to rate the quality of their last sleep episode, current daytime sleepiness, and current level of tension. All measures were reported on 8-point Likert scales of 0 (extremely bad sleep quality, not sleepy at all, and not tense at all) to 7 (extremely good sleep quality, extremely sleepy, and extremely tense). Beginning on day 15, daily surveys also included a question about whether earbuds were used during the last sleep episode (Multimedia Appendix 2).

#### Psychomotor Vigilance Testing

After every overnight shift, each participant was approached by a trained study research assistant to complete a 3-minute psychomotor vigilance test (PVT, PVT Research Tool, Texas A&M University System CSE) [13]. This test was conducted on portable tablet devices requiring participants to rapidly tap a circle as it appeared on a tablet screen, and the reaction time for each tap was measured. Participants were able to decline participation in the daily PVT if clinical demands required their attention.

#### 6-Month Follow-up

After the study period, participants were allowed to keep their earbuds for their own personal use. At 6 months after study completion, participants were sent an electronic survey asking about sleep aid use since study completion, including earbud use. For participants still using earbuds, additional questions about when and how they most commonly used the device since the study period were included.

### Data Management and Statistical Analyses

Study data were collected and managed using REDCap electronic data capture system sponsored and hosted at our institution. Data were deidentified prior to analysis and analyzed using Stata (version 16; StataCorp). Frequencies and percentages were used to summarize binary and categorical variables. Mean (SD) or median (IQR) values were used to summarize time variables and Likert scale data.

Changes in quality of the last sleep episode, current daytime sleepiness, and current level of tension in control and intervention periods were assessed using a linear mixed effects regression model with a participant-specific random intercept. In the postimplementation period, we only included data collected on days when participants reported using the earbuds. For PVT data, each participant’s mean reaction times pre- and postintervention period were directly compared. Six-month follow up responses were qualitatively assessed.

## Results

### Participant Demographic Characteristics

**Figure 1:**
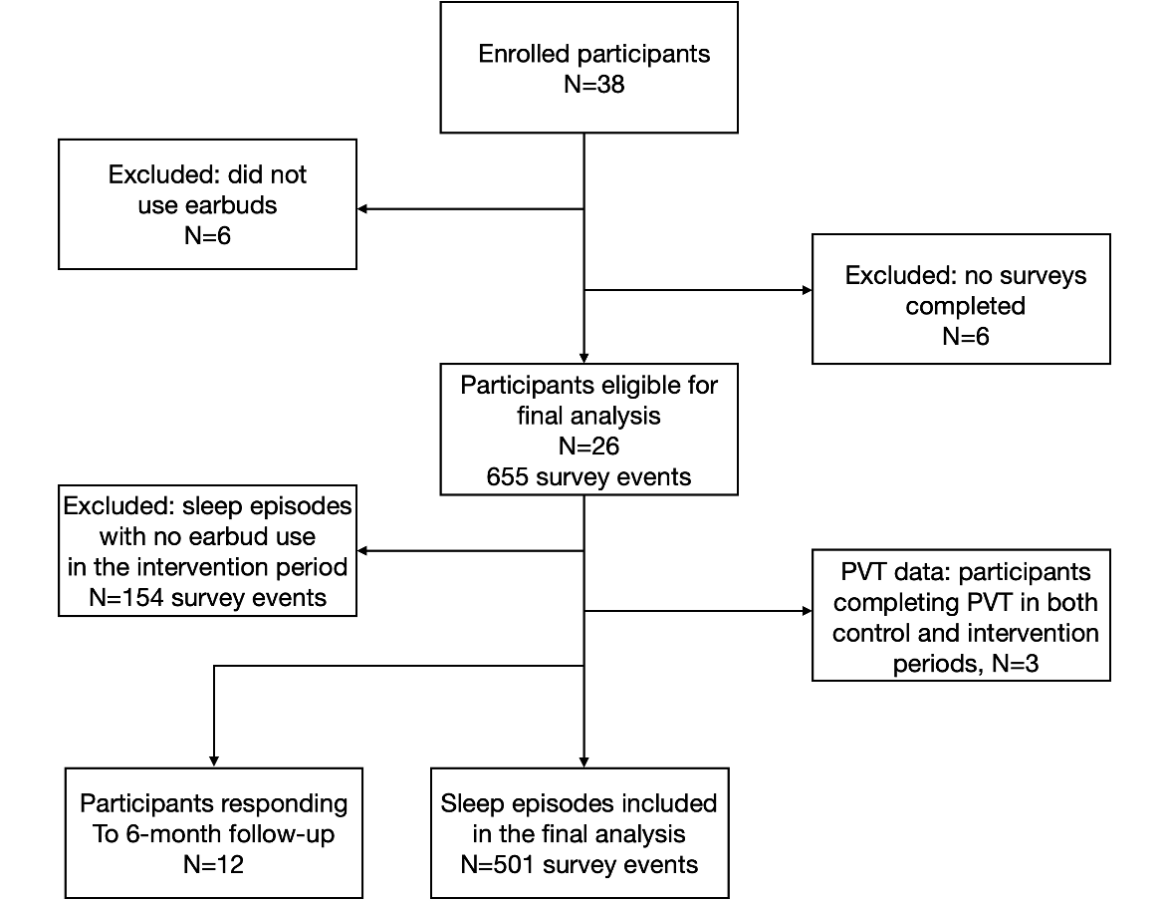
Study design flow chart. PVT: psychomotor vigilance test.

Of 58 invited participants, 38 participants enrolled in the study. Of these, 6 participants who did not complete any daily surveys and 6 who participated in surveys but never used the earbuds were excluded. The remaining 26 participants completed a total of 655 daily sleep surveys. We further excluded surveys taken on postintervention days when participants reported not using earbuds, resulting in 501 daily surveys being included in final analysis (Figure 1). Participants included in final analysis represented resident physicians from all 4 postgraduate years. In total, 27% of participants included in the final analysis were female, and their ages ranged from 25 to 35 years (Table 1).

**Table 1:** Participant demographic characteristics (N=26).

Variables			Participants
**Sex, n (%)**			
	Male	19 (73.1)	
	Female	7 (26.9)	
**Age (years), n (%)**			
	20-25	0 (0)	
	25-30	12 (46.1)	
	30-35	14 (53.9)	
	**>**35	0 (0)	
**Training year, n (%)**			
	PGY1	5 (19.2)	
	PGY2	9 (34.6)	
	PGY3	7 (26.9)	
	PGY4	5 (19.2)	
**Baseline average sleep or tension measures over the 4 prior weeks, median (IQR)**			
	Sleep quality	5 (4-6)	
	Sleepiness	4.5 (4-5)	
	Tension	5 (3-5)	
**Baseline sleep and aid use, n (%)**			
	Blackout curtains	16 (61.5)	
	Eye mask	8 (30.8)	
	Earplugs	6 (23.1)	
	Weighted blanket	1 (3.8)	
	White noise	9 (34.6)	
	Pharmacological sleep aid^a^	9 (34.6)	
	Other	1 (3.8)	
	None of the above	2 (7.7)	

^a^Pharmacological sleep aid refers to oral medications such as melatonin, benzodiazepines, zolpidem, or diphenhydramine.

### Earbud Use

**Figure 2:**
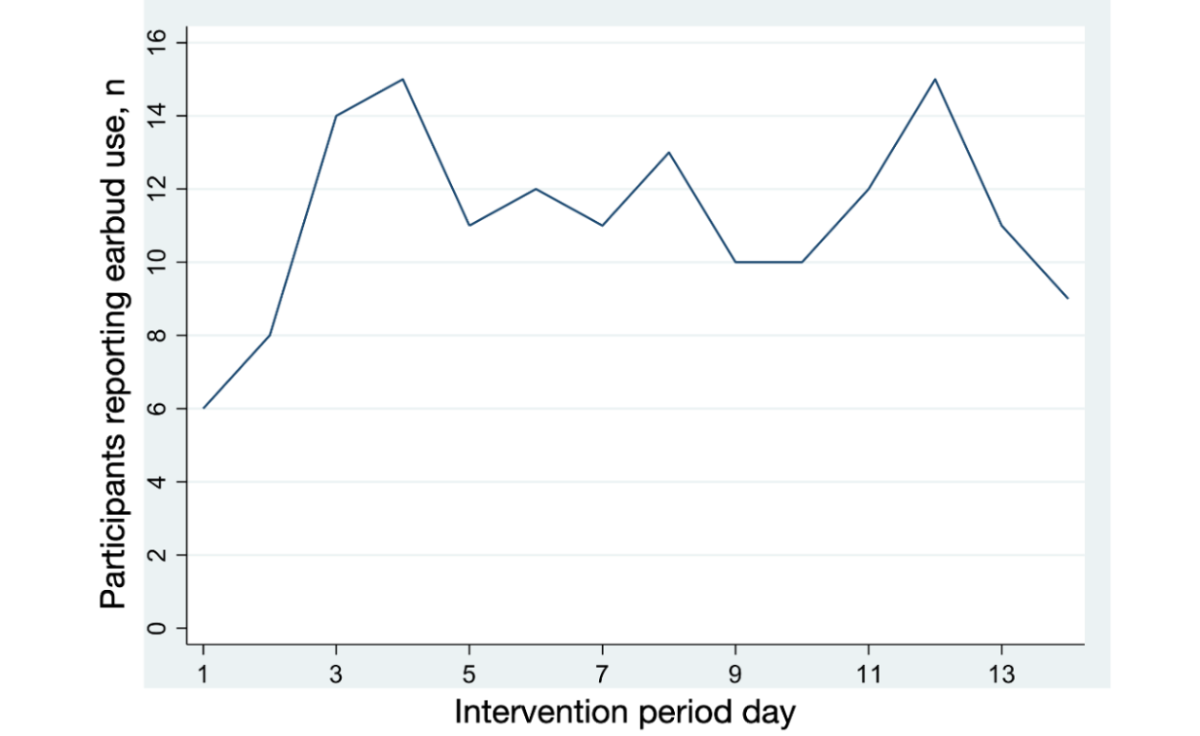
Reported earbud use over the intervention period.

Among participants who reported using earbuds at least once, the frequency of use varied. Of 14 days when earbuds were available, participants used earbuds for a median of 5 (IQR 2-9) days. The lowest earbud use occurred on day one of the intervention, with 6 of 26 participants reporting use, trending upward until day 4 when 15 of 26 participants reported using earbuds, and remaining generally stable with a slight downward trend in use toward the end of the study (Figure 2). Participants who reported using earbuds for ≥7 of the 14 days did not have significantly different baseline prestudy 4-week sleep scores than those who reported using earbuds between 1 and 6 days (mean score 5.2 vs 4.8, *P*=.34).

### Sleep Quality, Daily Sleepiness, and Tension

**Figure 3:**
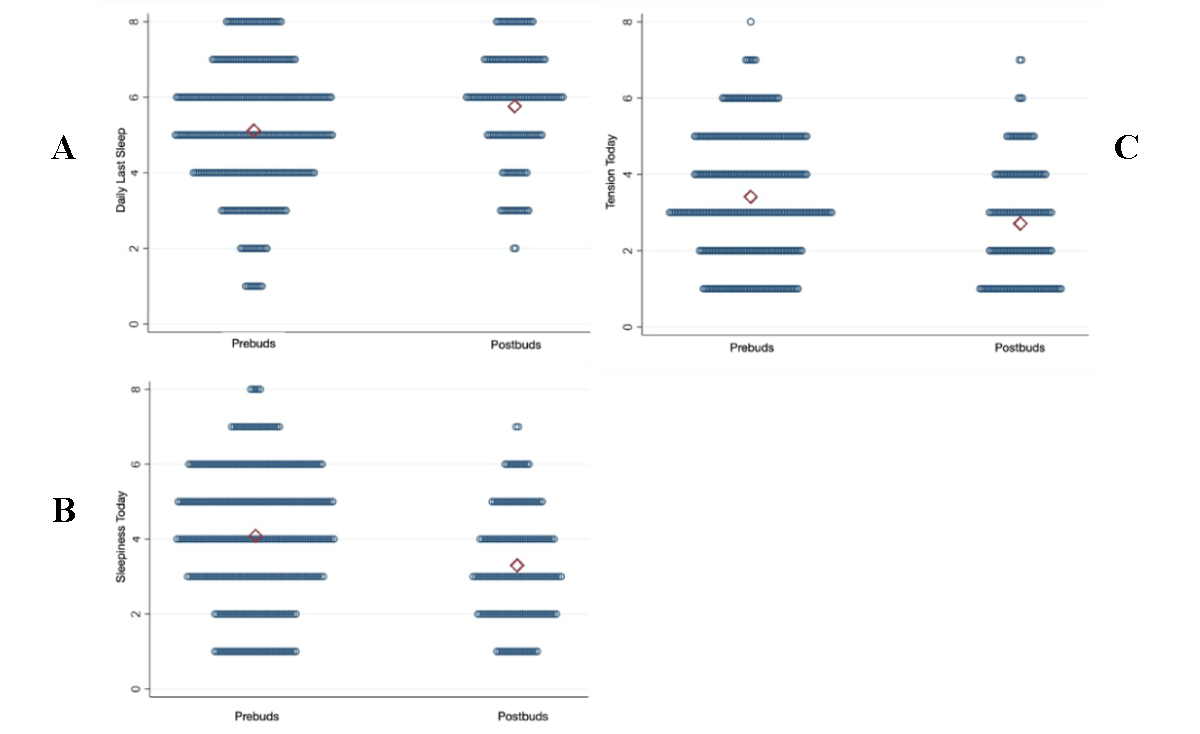
Self-rated last nights’ sleep quality (A), daily sleepiness (B), and daily tension (C) on days with earbud use during the prior sleep period.

Participants’ self-reported last sleep episode quality, daily sleepiness, and daily tension had improving trends in the postintervention period (Figures 3A-C). On days when residents used earbuds, on Likert scales of 0 to 7 points, previous nights’ sleep quality increased by 0.5 points (*P*<.001, 95% CI 0.23-0.80), daily sleepiness decreased by 0.6 points (*P*<.001, 95% CI –0.90 to –0.34), and total daily tension decreased by 0.5 points (*P*<.001, 95% CI –0.81 to –0.32) using linear mixed regression models. In the subset of participants who reported below-median sleep scores before the intervention, the beneficial effects of using earbuds were amplified.

### Post–Night Shift Reaction Time and Alertness

In total, 7 post–overnight shift residents completed a total of 12 PVT tests during the intervention period. A total of 6 completed PVTs were linked to participants who reported no earbud use and were thus excluded from the final analysis. Ultimately, 3 participants both used the earbuds and completed PVTs in both the control and intervention periods. Data from these participants comparing pre- and postearbuds mean reaction time showed no significant differences; however, this study was underpowered to detect an effect of earbud use on PVT.

### 6-Month Follow-up

We received responses from 12 (46%) participants. Of the 12 respondents, 5 reported ongoing earbud use; all of whom reported use on less than 25% of all sleep episodes since study completion. Participants cited limited sound options, uncomfortable fit, and forgetting to use the device as barriers to further use. There were no notable differences in self-reported baseline sleep aid use and 6-month follow up sleep aid use.

## Discussion

### Principal Findings

We report that adding a noise-masking earbud, Bose SleepBuds, to a sleep routine can foster significant improvements in shift workers’ self-reported sleep quality, sleepiness, and tension over baseline sleep habits. Participants in this sample noted statistically significant improvements following use of earbuds, and minimal safety concerns were identified. In health care providers, poor sleep contributes to decreased alertness and performance, which adversely affects patient care. Sleep-deprived shift workers demonstrate decreased mood, and professional fulfillment, emotional exhaustion, and increased rated of burnout [10,14-18].

Sleep and wake disturbances are common among shift workers and can produce a primary circadian rhythm disorder called “shiftwork sleep disorder” (SWSD) characterized by insomnia, excessive sleepiness, and significant sleep loss [6,19,20]. Sleep problems are associated with debilitating chronic physical and mental health disease. In addition to excessive sleepiness, sleep deficiency and SWSD are associated with critical short- and long-term health concerns. Sleep deficiency in shift workers has been associated with obesity, type 2 diabetes, stroke, multiple types of cancers, and cardiovascular disease [21-24].

To combat shift work–associated sleep loss, shift workers may develop self-designed strategies to assist with sleep. One common approach is to alternate between the use of pharmacological stimulants and sedatives to achieve wakefulness or sleep as needed. Staying awake and alert during shift can require aids such as caffeine or nicotine [25,26]. To accelerate the onset of sleep, over-the-counter supplements such as melatonin, prescription sleep aids such as benzodiazepines, or other sedative-hypnotic medications (eg, so-called “Z-drugs” such as zolpidem) are also often used [26-28]. Despite the fact that alcohol consumption worsens sleep overall, postshift alcohol use is another common strategy among shift workers to achieve sleep, a strategy that has been associated with binge drinking disorder [29,30]. Each of these strategies demonstrate variable efficacy and can have significant off-target, often deleterious, health effects, development of compulsive use consistent with addiction, and poor patient care.

Participant adherence and uptake in the intervention was one challenge faced in this study. When considering wellness interventions, in addition to intervention efficacy, institutions must also consider factors such as participant interest and adherence and cost-benefit ratios of a given intervention. Here, roughly two-thirds of the approached participants elected to enroll in the study in which they were receiving sleep aid devices free of charge. It is possible this number may serve as a proxy for interest in such nonpharmacological sleep aid devices or in sleep interventions in general. Direct assessment of shift worker interest in nonpharmacological sleep interventions, such as this one, are needed. Interestingly, a larger proportion of males than females enrolled in the study despite the fact that the group originally solicited was roughly even in terms of sex. This may suggest that an electronic sleep device such as noise-masking earbuds are more attractive to males than to females as an intervention. Further analysis of the most likely users may be helpful in targeting sleep interventions to users who may benefit from or adhere most to them. Finally, cost-benefit ratio is often a consideration when implementing wellness initiatives. Though the particular noise-masking earbuds studied here have a relatively high price point for the general public, targeting these devices to participants who may have the highest uptake, adherence, and physiological outcomes may result in a favorable cost-benefit ratio for organizations. Further work is needed to more carefully target sleep interventions such as the earbuds studied here.

Shift workers experience striking rates of workplace burnout; yet, occupational interventions targeting sleep problems are often lacking compared to other wellness initiatives [12]. The importance of this work, therefore, is that noise-masking earbuds may improve several metrics of sleep quality without the threat of problematic substance use, medication adverse events, or pharmaceutical misuse. Although we identified several beneficial effects of the noise-masking earbuds on self-reported sleep and tension, alertness testing via PVT was underpowered to detect an effect in our study population. Because health care professionals are particularly vulnerable to the deleterious effects of sleep loss and SWSD is prevalent in up to 30% of the general population, our findings, while limited, have broad therapeutic potential in multiple industries where shift work is common, including medicine.

### Limitations

This was a single-arm study with a small sample size, and variable intervention adherence, all of which affected the analytical power of this study. Further efficacy should be evaluated in a randomized controlled trial comparing earbud use to a matched control. Here, we focused on resident physicians as representatives of the health care shift worker population. This population tends to be fairly young (between 25-35 years old), and may be more willing to use new technology than shift workers as a whole. While approximately one-third of resident shifts are overnight, night shifts tend to be clustered and are not evenly distributed over a 28-day period. Thus, typical resident schedules are somewhat suboptimal for a single-subject design where subjects serve as their own controls across 28-days. The PVT data collection was limited given the smaller number of data points across control and intervention periods for individual participants. Future solutions may include recruiting study participants such as nocturnists, who are physicians who work a higher proportion and more consistent distribution of night shifts, other groups who work exclusively overnight, or participants with clinically diagnosed SWSD. Increasing study population size or extending the study period by multiple months would likely enhance our data points and improve study power.

Variable intervention adherence also affected our findings. Manually filling out daily surveys and PVT testing after overnight shifts can be burdensome to participants. Participants may have forgotten to complete surveys after receiving the SMS text message reminder. Future studies will consider alternative data collection strategies that may present less of a barrier, such as automatic data collection on sleep quality via wearable fitness trackers or cellular telephones. These strategies may also provide more objective measures of sleep quality as opposed to self-reporting. In terms of inconsistent device use, participants cited a desire for additional sound options including selectable music, improved device fit, and needing reliable reminders to improve intervention adherence. Addressing these factors will be crucial to designing maximally effective digital therapies for sleep deprivation in the future.

The potential for sustained adherence is an important consideration in any wellness intervention. Here, at 6-month follow up, <50% of respondents reported ongoing earbud use beyond the study period. Six-month follow-up responses were collected from only 46% of original participants; thus, the limited response rate likely impacts our ability to draw firm conclusions from these data. Subjectively, however, reasons commonly cited for limited ongoing use included poor fit, limited audio tracks, and forgetting to use the earbuds. The fit and audio selection can be addressed by manufacturers to make such products more attractive to users. Habit formation and establishing automated behaviors are key to implementing sustainable health and wellness interventions. Data from nutritional wellness interventions suggest habits can take more than 50 days to reach automaticity [31]. Regarding forgetting to use the device, it is likely our study period was not long enough to promote habit formation. Prolonging the intervention study period with frequent reminders to use the device, or instructing participants to place the device in a convenient, easily accessible location that will remind and reinforce use can be helpful. Further, time-based cues such as reminders on cellular telephones can help establish behaviors while habit formation is taking place [31].

As a pilot study, the aim of this work was to assess uptake, user receptiveness, and perceived effect of a nonpharmacological sleep intervention. Important next steps for future studies include evaluating objective measures of sleep and mood, physician burnout among earbud users, as well as potential changes in clinician performance and patient outcomes such as physician errors or return visit rates. While participant experience is an important component in evaluating the effect of a wellness intervention, objective outcomes studies will help corroborate the utility of implementing such interventions on an institutional level.

### Comparison With Prior Work

While electronic devices aimed at addressing sleep problems do currently exist, few have robust data supporting their efficacy. Most common devices addressing poor sleep include fitness trackers or mobile apps to track sleep quality, but these rarely offer therapeutic interventions [32,33]. Existing digital therapies for poor sleep include app-based meditation or relaxation guides, and app-guided cognitive behavioral therapy [34,35]. Though moderately effective in some populations, these interventions are often targeted at addressing chronic sleep loss and insomnia, are subject to intervention fatigue, and do not address acute sleep problems and circadian rhythm disruption common in post–night shift health care workers.

Devices supplying white noise are commonly marketed as sleep aids. Despite broad commercial penetrance, evidence for the efficacy of continuous white noise on sleep onset, latency, or quality is inconsistent [36-39]. Many factors likely play into the effect of ambient sounds on sleep, including but certainly not limited to the type of sound, the ability to block out alternative ambient noises, and sound volume among other factors. The SleepBuds technology used in this study included first-generation devices that provide noise-masking technology to reduce ambient auditory distractions with optional relaxing sounds. Given our promising results, future work can explore the effect of subsequent-generation devices with enhanced functionality targeted at relaxation. This work will both assess the efficacy of this digital intervention, as well as contribute to the existing body of work on the therapeutic effect of ambient noise on sleep overall.

### Conclusions

Sleep loss is a public health crisis that disproportionately affects the physical and mental health of shift workers. The recent emphasis on support programs aimed at improving employee wellness and decreasing burnout is not reflected in interventions aimed at improving sleep. Our data demonstrate the efficacy of a digital intervention addressing sleep disturbance. This intervention is of low risk and is feasible in a complex cohort such as medical resident physicians. While our target population was health care shift workers, extension of our findings to other industries and cohorts is reasonable. Our work prepares for additional studies to improve employee health and wellness through effective, generalizable, and scalable sleep interventions.

## Appendix

Multimedia Appendix 1 A. Entrance survey completed by all participants. B. Exit survey completed by all participants.

Multimedia Appendix 2 Daily surveys delivered to all participants via text message during the control period (A), and intervention period (B).

## Abbreviations

ED: emergency department
EM: emergency medicine
IRB: institutional review board
PVT: psychomotor vigilance test
SWSD: shiftwork sleep disorder
