# Supplementary material for: The Effect of Noise-Masking Earbuds (SleepBuds) on Reported Sleep Quality and Tension in Health Care Shift Workers: Prospective Single-Subject Design Study
Source: JMIR Form Res. 2022 Mar 22;6(3):e28353. doi: 10.2196/28353 (PMC8984824; doi:10.2196/28353)
Supplement: Multimedia Appendix 1 [file formative_v6i3e28353_app1.docx]

**Appendix 1.** A. Entrance survey completed by all participants. B. Exit survey completed by all participants.

1. SleepBuds Study - Enrollment Survey

Demographics:

1. Name
2. Phone number to receive daily text messages
3. Date of birth
4. Gender
5. Residency year
6. Last rotation
7. Current rotation

Current Sleep Aids:

1. Current use of sleep aids:
   1. Blackout Curtains
   2. Eye mask
   3. Earplugs
   4. Weighted blanket
   5. White noise
   6. Pharmacological sleep aid
   7. Other (describe below)
   8. None of the above

Current Sleep Quality:

9. How would you evaluate your average sleep over the last 4 weeks? (Likert 0-7, extremely bad to extremely good)

10. How sleepy have you felt on an average day over the last 4 weeks? (Likert 0-7, not sleepy at all to extremely sleepy)

11. How tense do you feel on an average day over the last 4 weeks? (Likert scale 0-7, not tense at all to extremely tense)

1. SleepBuds Study – Exit Survey
2. Name
3. Do you use any of the following as sleep aids? (select all that apply)
   1. Blackout Curtains
   2. Eye mask
   3. Earplugs
   4. Weight blanket
   5. White noise machine
   6. Pharmacologic sleep aid
   7. None of the above
4. Have you decreased your use of these sleep aids over the last 4 weeks? (Likert 0-7, significant decreased to no change)

Current Sleep Quality:

1. How would you evaluate your last sleep period? (Likert 0-7, extremely bad to extremely good)
2. How sleepy do you feel today? (Likert 0-7, not sleepy at all to extremely sleepy)
3. How tense do you feel today? (Likert scale 0-7, not tense at all to extremely tense)
4. Did you use the SleepBuds during your last sleep period? (yes, no)
5. What was the most recent shift period you worked in the last 24 hours? (Day shift, Afternoon/Evening shift, Overnight shift, Did not work in the last 24 hours)
