# Supplementary material for: The Effect of Noise-Masking Earbuds (SleepBuds) on Reported Sleep Quality and Tension in Health Care Shift Workers: Prospective Single-Subject Design Study
Source: JMIR Form Res. 2022 Mar 22;6(3):e28353. doi: 10.2196/28353 (PMC8984824; doi:10.2196/28353)
Supplement: Multimedia Appendix 2 [file formative_v6i3e28353_app2.docx]

**Appendix 2.** Daily surveys delivered to all participants via text message during the control period (A), and intervention period (B).

1. SleepBuds Study - Daily Sleep Survey: (study period: Days 1-14)

1. How would you evaluate your last sleep period (Likert 0-7, extremely bad to extremely good)

2. How sleepy do you feel today (Likert 0-7, not sleepy at all to extremely sleepy)

3. How tense do you feel today (Likert 0-7, not tense at all to extremely tense)

4. What was the most recent shift period you worked in the last 24 hours? (Day shift, Afternoon/Evening shift, Overnight shift, Did not work in the last 24 hours)

1. SleepBuds Study - Daily Sleep Survey (study period: Days 15-28)

1. How would you evaluate your last sleep period (Likert 0-7, extremely bad to extremely good)

2. How sleepy do you feel today (Likert 0-7, not sleepy at all to extremely sleepy)

3. How tense do you feel today (Likert 0-7, not tense at all to extremely tense)

4. Did you use the Sleepbuds during your last sleep period? (yes, no)

5. What was the most recent shift period you worked in the last 24 hours? (Day shift, Afternoon/Evening shift, Overnight shift, Did not work in the last 24 hours)
